# Strategic inter-cropping for saline agriculture: quantifying the impact of *Medicago sativa–Festuca arundinacea* ratios on soil properties and crop performance

**DOI:** 10.3389/fpls.2025.1737387

**Published:** 2025-12-19

**Authors:** Yuchen Sun, Jiayu Shi, Xindi Liu, Li Zhao, Guofeng Yang, Qibo Tao, Shangzhi Zhong, Qingping Zhang, Fuhong Miao

**Affiliations:** 1 College of Grassland Science, Qingdao Agricultural University Qingdao, Qingdao, China; 2 Shandong Key Laboratory for Germplasm Innovation of Saline-alkaline Tolerant Grasses and Trees, Qingdao, China; 3 Agricultural Research Institute of Saline and Alkaline Land of Yellow River Delta, Dongying, China; 4 College of Agriculture and Forestry Science, Linyi University, Linyi, China

**Keywords:** forage inter-cropping, forage productivity, intercropping competitiveness, soil physicochemical properties, sustainable development

## Abstract

Soil salinization is an abiotic stress that hinders crop growth, agricultural productivity, and environmental protection. In this study, alfalfa (*Medicago sativa*) and tall fescue (*Festuca arundinacea*) were sown in seven inter-cropping ratios, with monocultures as controls to explore the effects of inter-cropping grasses on yield, water-soluble salt content, pH, and total nitrogen in saline-alkali land, and to establish whether inter-cropping can alleviate salinity and alkalinity. In addition, this study aimed to screen and identify the best alfalfa and tall fescue inter-cropping ratio. The results revealed that (1) Alfalfa and tall fescue had the best productivity and the highest crude protein content at an inter-cropping ratio of M6F4, M7F3, and M8F2, respectively. Besides, inter-cropping improved the land-use efficiency of saline land by altering the plant stem-leaf ratio to adapt to the resource competition. (2) Alfalfa and tall fescue inter-cropping at M3F7, M4F6, and M7F3 decreased the 21% soil salt and 7.8% pH and increased the 34.7% total nitrogen content. (3) Correlation analysis revealed significant correlations among soil salt content, pH, nitrogen, inter-cropping yield, stem-leaf ratio, and plant competition rate. These findings indicate that inter-cropping alfalfa and tall fescue in the ratio M6F4, M7F3, and M8F2 best improves the utilization efficiency of saline land.

## 1 Introduction

Salinization results in land degradation, crop yield reduction, and restricted sustainable development of agriculture globally (Nan et al., 2021). The Yellow River Delta, a typical coastal alluvial plain in northern China and one of the most severely salinized regions nationwide, is representative of global coastal saline-alkali ecosystems shaped by seawater intrusion and groundwater dynamics (Gao et al., 2018). However, with the prolonged invasion of seawater, the rise of the deep groundwater table, and the impact of climate change, soil salinization in this delta has been aggravated over the past 20 years, seriously limiting local agricultural productivity and economic development (Fan et al., 2011; Zheng et al., 2017; Zhang and Wang, 2021). Inter-cropping can improve land utilization efficiency, especially saline land, turning it into an important reserve cultivated land resource, a significant, sustainable development approach for agriculture saline land.

Groundwater table depth and salinity are key factors influencing the formation and development of saline-alkali land, where a rising groundwater table and high salinity collectively increase soil salinity (Guan et al., 2001). However, these lands can be reclaimed by adopting saline-alkali-favorable farming methods, including managing saline-alkaline soil and water and cultivating salt-tolerant crops (Katarzyna et al., 2022). For example, strategies such as tillage, straw mulching, and irrigation reduce soil salinity by reducing soil water evaporation and leaching surface (Ahmad et al., 2022).

In areas where salinity is affected, excess salinity can lead to water intake and nutrient uptake, which can lead to a 20%-45% decrease in yield (Muhammad et al., 2024). Under abiotic stress, plants will show a variety of response mechanisms, including a series of measures such as regulating osmotic pressure and ion stability. When plants are subjected to saline-alkali stress, they will regulate solute accumulation in cells, which helps to maintain cell balance and cope with swelling stress (Ashraf et al., 2021). For example, alfalfa showed no growth inhibition under mild and moderate salt stress, and obvious growth inhibition under severe salt stress and alkali stress (Zhang et al., 2024).

Moreover, inter-cropping crops with forage enhances productivity, yield stability, and soil quality—thereby increasing economic benefits and further improving soil fertility (Wang et al., 2020). Studies have shown that double-season crops with high planting intensities can improve yield and land use efficiency (Miao et al., 2019). Crops compete for light, heat, water, fertilizer, and other resources during inter-cropping, and improves their utilization efficiency. Under the same land area, the yield from inter-cropping exceeds that of single cropping. Additionally, previous studies have shown that changing the proportion of crops planted, or appropriately increasing the strip width between crops, can promote crop growth and greatly increase yield. Therefore, inter-cropping not only improves the yields, but also enhances the soil health and nutrition (Snyder et al., 2020). For example, in cotton (*Gossypium*) and alfalfa (*Medicago sativa*) inter-cropping system, the salt accumulation and soil pH were reduced, increasing the soil desalination capacity (Liang and Shi, 2021). Cotton and alfalfa were intercropped in saline-alkali land, and cotton and alfalfa were intercropped evenly between films. The water retention rate of 0-40cm soil layer increased by 29.80%, the salt inhibition rate reached 125%, and the yield of seed cotton increased by 7.74% compared with the control (Xin et al., 2010).

In addition, alpine area-grown crops in the Suaeda salsa maize inter-cropping system transfer more sodium to the roots, reducing the soil salt content (Wang et al., 2022). However, the effectiveness of intercropping is modulated by multiple factors, including interspecific row ratio, planting density, and resource allocation. For instance, in an alfalfa - brome inermis intercropping system configured with a 2:1 row ratio (2 rows of alfalfa alternating with 1 row of brome) and a total planting density of 45 plants/m², the total forage yield was significantly higher—28% greater than that of alfalfa monoculture and 35% higher than that of brome monoculture (Yi et al., 2022; Fuhong et al., 2023).

Therefore, perennial pasture use for inter-cropping can improve the economic benefits and promote sustainable development of agriculture (Reynolds et al., 2021). Alfalfa is an excellent perennial forage with diverse functions, widely cultivated in saline lands worldwide. Studies have found that alfalfa reduces salt accumulation in the soil, promotes the reduction of sodium ions, and improves the condition of saline soils (Yang et al., 2025). Studies have confirmed that the prominent role of alfalfa in saline-alkali land improvement depends on two core salt tolerance mechanisms: first, its deep taproot system enhances salt leaching by accelerating water infiltration, and symbiotic rhizobium fixes 180–220 kg of nitrogen per hectare per year to improve soil fertility (Cangioli et al., 2025); Secondly, it actively excludes sodium ions in root cells by upregulating MsRCI2D/E gene expression, reducing cytoplasmic Na^+^ concentration by 35% (Lv et al., 2025).

On the other hand, tall fescue (*Festuca arundinacea*) is a cold-tolerant perennial grass with strong ecological adaptability used in pasture hay silage and turf production (Talukder et al., 2018). It is a high-yielding grass with a high nutritional value and salt tolerance. In addition, it exerts a certain improvement effect on saline land in coastal areas.

Including perennial crops in inter-cropping systems improves the yields (Bybee-Finley et al., 2023). However, different inter-cropping ratios may result in competitiveness between the intercropped species. Therefore, in this study, alfalfa and tall fescue were used to evaluate the changes in agronomic traits, total nitrogen (TN), pH, and water-soluble salt content during 2022-2023. Improve the use efficiency of saline-alkali land in the strip-shaped perennial forage intercropping system with different proportions, and at the same time, the corresponding land use efficiency, agronomic traits and soil physical and chemical properties of different forage grasses will promote the sustainable development of agricultural saline-alkali land.

## 2 Materials and methods

### 2.1 Experimental design

The experiment was performed in the Yellow River Delta agricultural high-tech industry demonstration zone of Shandong Province during 2022-2023 (E118°66′, N37°32′), a typical alluvial plain coast consisting of soil saline land. The area experiences a warm temperate monsoon continental climate, with an average rainfall of 668mm in the past two years. Rainfall mainly occurs from May to September. The average temperature is 5°C in the total year. The soil pH is 8.7, with a 2.1 dS/m salinity. Thus, the soil is classified as mildly saline-alkali alluvial soil. The saline soil in this area is widely distributed, with many types.

There were seven intercropping ratios between alfalfa and tall fescue. The intercropping ratio of alfalfa and tall fescue is M2F8 to M3F7, M4F6, M5F5, M6F4, M7F3, M8F2, where M stands for alfalfa, F stands for tall fescue, and the numbers after the letters represent the number of planted rows. Each plot has 20 rows, distributed according to the intercropping proportion, with a row spacing of 20cm. For example, M2F8 stands for alfalfa 2 rows of tall fescue 8 rows, and plant another 2 rows of alfalfa and 8 rows of tall fescue. Pure stands of alfalfa (sMed) and tall fescue (sFes) were used as controls. Therefore, the experiment consisted of nine treatments, each replicated three times with each plot size of 4 m × 8 m (32 m²). The trial employed a completely randomized block design in which, within each block, nine treatments were randomly assigned to a single plot, with all adjacent plots separated by isolated rows 2 m wide. These segregated rows help prevent cross-contamination of water, nutrients and salts between treatments, mitigate edge effects, and avoid interference from competition between plots. The row spacing and sowing rate of inter-cropping crops were the same as that of the monoculture. The sowing rate was 15kg/ha for alfalfa and 40 kg/ha for fescue. The basal fertilizer CO (NH_2_)_2_ (15%)-P_2_O_5_ (15%)-K_2_O (15%) was also applied at a fertilization rate of 571.035 kg/ha. Other conventional field management practices include routine watering, weed control, and pest control according to the local climate and soil habits.

### 2.2 Plant and soil sampling

The study was conducted from April 2022 to May 2023, with alfalfa and tall fescue sowing dates in late April 2022. Alfalfa and tall fescue were sampled in June 2022 and May 2023, with plant and soil samples collected simultaneously. Three 2m × 0.5 m quadrats were randomly set up along the diagonal line in each plot, and the distance between the quadrats was not less than 0.5 m from the plot boundary to avoid the edge effect. All plants in the plot were harvested, and the fresh weight of alfalfa and tall fescue was measured respectively. The average value of the three quadrats was taken as the single species biomass of the plot. From each plot, 500g of fresh samples were carried to the laboratory, where they dried in the oven at 105 °C for 40 minutes and then at 65°C for over 12 hours. Soil samples from 0-30cm were collected at three random points in each plot, and were divided into three sections of 10cm each. The sections were naturally dried for 7 days, ground with a mortar, and then sieved through a 2mm and a 0.1mm sieve (Laika et al., 2024).

### 2.3 Measurement of plant growth indexes and soil chemical analysis

The stem-to-leaf ratio of 5–10 plants in random sampling per plot was determined using the following formula:


$$\text{stem}-\text{to}-\text{leaf ratio}=\frac{\text{Stem weight}}{\text{Leaf weight}}\;$$

Land equivalent ratio (LER): Competitiveness of crops to resource utilization in inter-cropping system. If LER > 1, it indicates some advantages in inter-cropping (Li et al., 1999).


$$\text{LER}=\frac{{\text{Y}}_{1\text{i}}}{{\text{Y}}_{1}}+\frac{{\text{Y}}_{2\text{i}}}{{\text{Y}}_{2}}$$

Competition rate (CR): CR measures the competitiveness of inter-cropped crops and evaluates the utilization efficiency of crop resources. CR makes up for the defect that LER fails to consider the planting ratio, effectively measuring the competitiveness of crops (Dhima et al., 2007).


$${\text{CR}}_{1}=\left(\frac{{\text{Y}}_{1\text{i}}}{{\text{Y}}_{1}}\times \frac{{\text{Y}}_{2}}{{\text{Y}}_{2\text{i}}}\right)\times \frac{{\text{Z}}_{2\text{i}}}{{\text{Z}}_{1\text{i}}}$$

$${\text{CR}}_{2}=\left(\frac{{\text{Y}}_{2\text{i}}}{{\text{Y}}_{2}}\times \frac{{\text{Y}}_{1}}{{\text{Y}}_{1\text{i}}}\right)\times \frac{{\text{Z}}_{1\text{i}}}{{\text{Z}}_{2\text{i}}}$$

Where Y1 and Y1i represent the monoculture and inter-cropping yields of alfalfa, and Y2 and Y2i the monoculture and inter-cropping yields of tall fescue, respectively. Z1i and Z2i are the planting ratios of alfalfa and tall fescue in the inter-cropping system, respectively.


RFV%=[(120/NDF)×(88.9-0.779×ADF)]/1.29

Relative Feeding Value (RFV): RFV is based on Digestible Dry Matter (DDM) and Dry Matter Intake (DMI). For roughages, DDM and DMI are derived from laboratory-measured Neutral Detergent Fiber (NDF) and Acid Detergent Fiber (ADF), respectively. The NDF and ADF analyses are conducted using the filter bag technique (Van Soest Method). After boiling with neutral detergent, the insoluble residue is neutral detergent fiber, mainly cell wall components, including hemicellulose, cellulose, lignin and silicate. The plant-based feed is treated with an acid detergent, and the remaining residue is acid detergent fiber, which includes cellulose, lignin and silicate. The residue after 72% sulfuric acid treatment of acid detergent fiber is lignin and silicate, and the residue after 72% sulfuric acid treatment is subtracted from the value of acid detergent fiber is the cellulose content of feed. The residue after 72% sulfuric acid treatment was ashed, and the part that escaped during the ashing process was the content of acidic washed lignin (ANKOM 2000i Fully Automated Fiber Analyzer) (Wei et al., 2024).

Crude protein (CP): The total nitrogen content of plants was determined by Vario EL III elemental analyzer (Elementar, Germany) based on the Dumas combustion method, and then multiplied by 6.25 to convert the crude protein content (Pošćić et al., 2016).

For the soil pH estimation, the soil soluble salts were extracted with water. Briefly, the soil and water were mixed at a ratio of 5:1, shaken for 20 minutes and then centrifuged for 5 minutes. Finally, the supernatant pH was determined using a pH meter (SevenExcellenceS400-Basic, China).

Subsequently, the supernatant was filtered and evaporated in a water bath. Next, the hydrogen peroxide was added to remove organic matter and evaporated to dryness for 1–2 hours before weighing. Finally, the total soil nitrogen content was measured with an elemental analyzer.

### 2.4 Statistical analysis

Yield, LER, CR, stem-leaf ratio, soil water-soluble salt content, soil pH, and soil total nitrogen (TN) were analyzed by one-way ANOVA using SPSS statistical software (version 22.0). In addition, Pearson correlation analysis was used to establish the correlations between indicators.

## 3 Results

### 3.1 Yield and yield components of plants with different inter-cropping ratios

**Figure 1:**
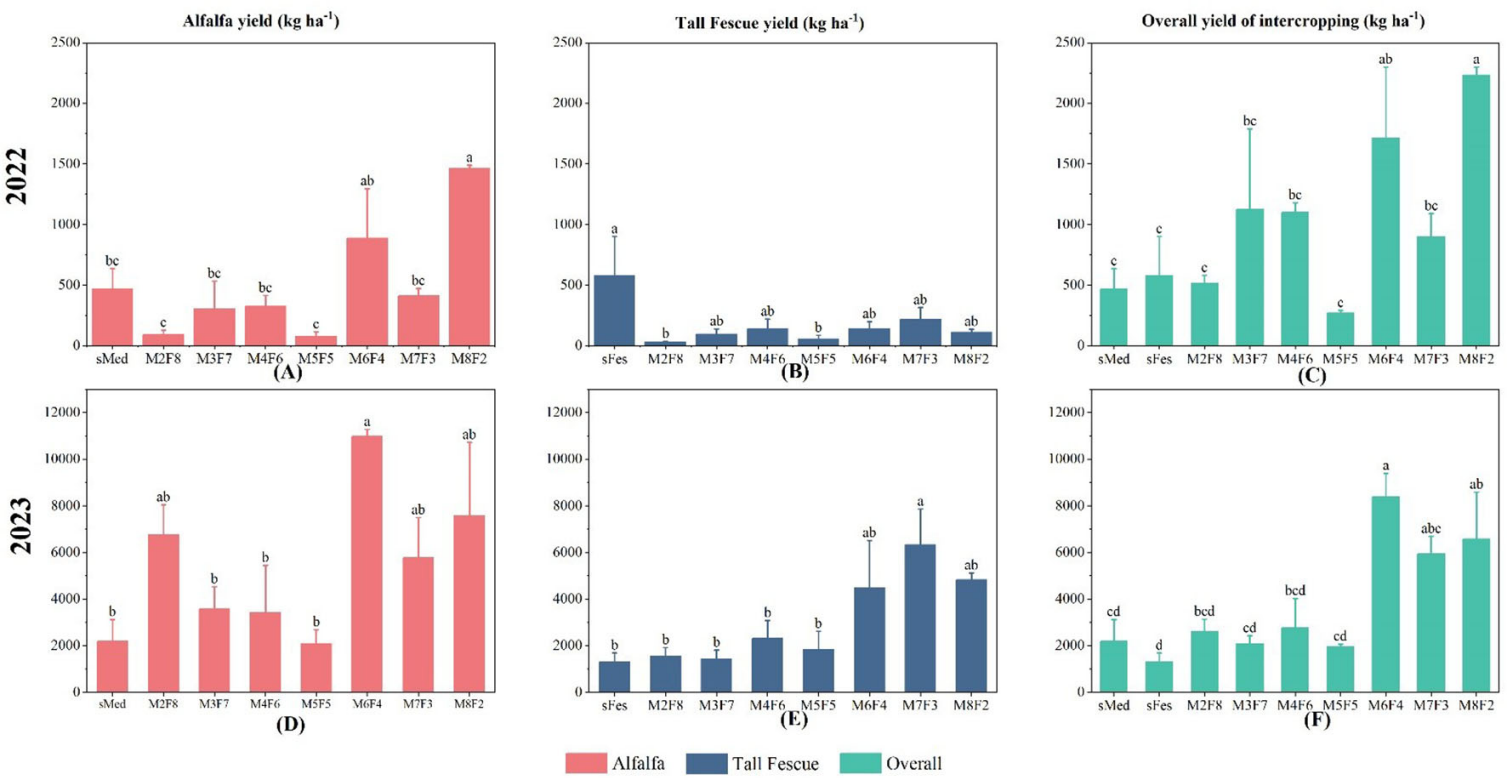
Alfalfa and tall fescue yields in 2022 and 2023 under different inter-cropping ratios and total inter-cropping yields. The significance level is P<0.05, and lowercase letters indicate significant differences between treatments. (Letter annotations and significance levels are the same in other images), sMed is unicast alfalfa, sFes is unicast tall fescue, M is alfalfa, F is tall fescue, and the numbers represent the ratio. For example, M2F8 represents 2 rows of alfalfa and 8 rows of tall fescue. **(A)** Alfalfa yields under different planting models in 2022. **(B)** Yield of tall fescue under different planting patterns in 2022. **(C)** Total alfalfa and tall fescue production in 2022 under different planting models. **(D)** Alfalfa yield under different planting models in 2023. **(E)** Yield of tall fescue under different planting patterns in 2023. **(F)** Total alfalfa and tall fescue production in 2023 under different planting models.

**Figure 2:**
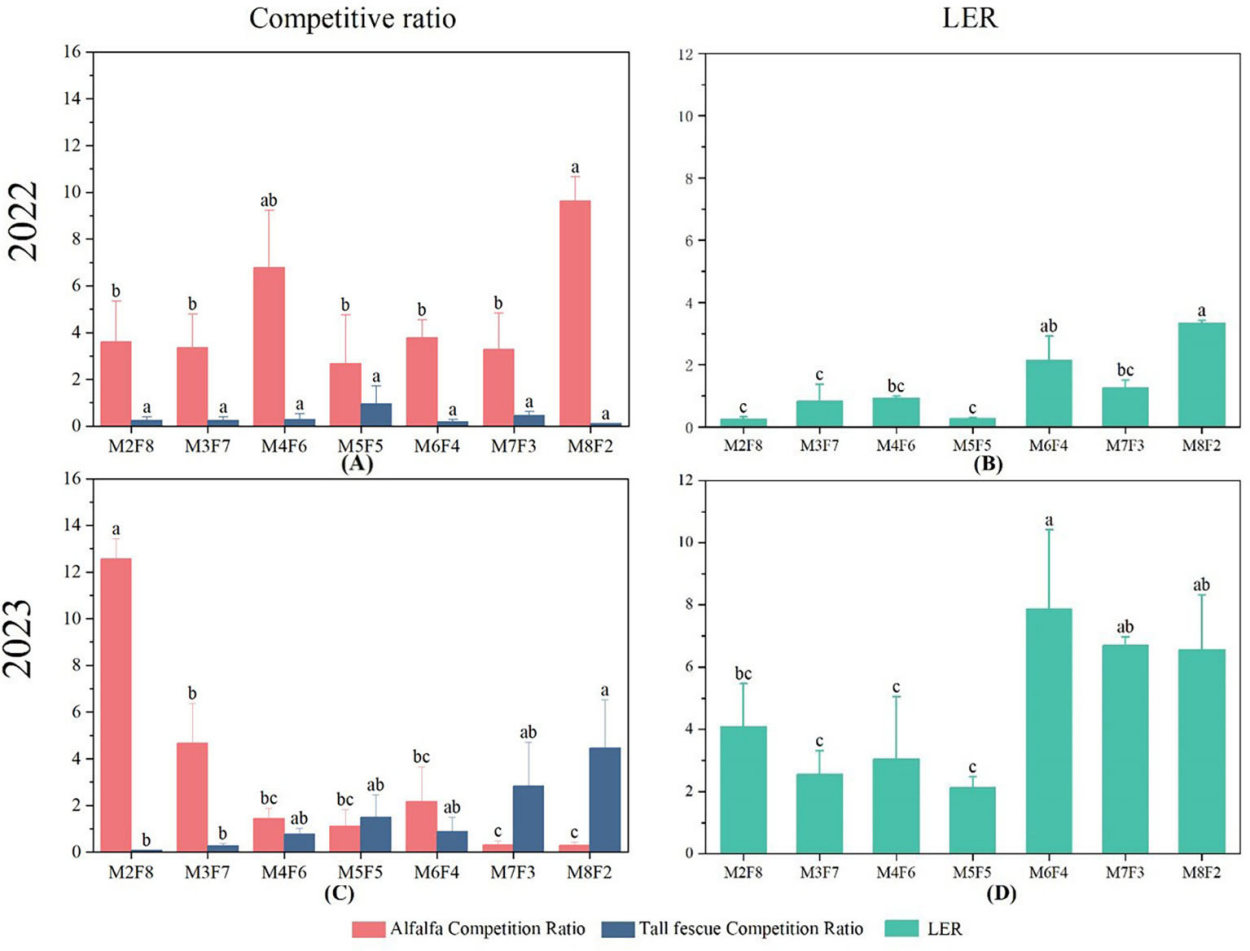
**(A)** CR (Competition Ratio) of alfalfa and tall fescue under different planting models in 2022. **(B)** LER (Land Equivalent Ratio) under different inter-cropping models in 2022. **(C)** CR of alfalfa and tall fescue under different planting models in 2023. **(D)** LER under different inter-cropping models in 2023.

In the first year (2022, **Figure 1**), the hay yields of M3F7, M4F6, M6F4, and M8F2 were 20-40% higher than those of other treatments, with M8F2 producing the maximum yields. In 2023, the hay yields of M6F4, M7F3, and M8F2 were 60-70% higher than those of other treatments, with M6F4 producing the highest yields. In both years, the LER of M6F4, M7F3, and M8F2 was greater than 1 (**Figure 2**). In addition, the yield of alfalfa and tall fescue in the different inter-cropping ratios was significantly different in 2022. The alfalfa yield in M8F2 was the highest, about 70% higher than in the other treatments. Notably, the tall fescue yield was lower than that in monoculture. In 2023, M6F4 had the highest alfalfa yield, approximately 79% higher than monoculture yields. In addition, M7F3, M8F2, M6F4, and M2F8 were significantly higher than other treatments. In tall fescue, M6F4, M7F3, and M8F2 yields were significantly higher than in other treatments, with the highest yields in M7F3, about 77% higher than in the monoculture.

2.2 The CR of plants with different inter-cropping ratios.

**Figure 3:**
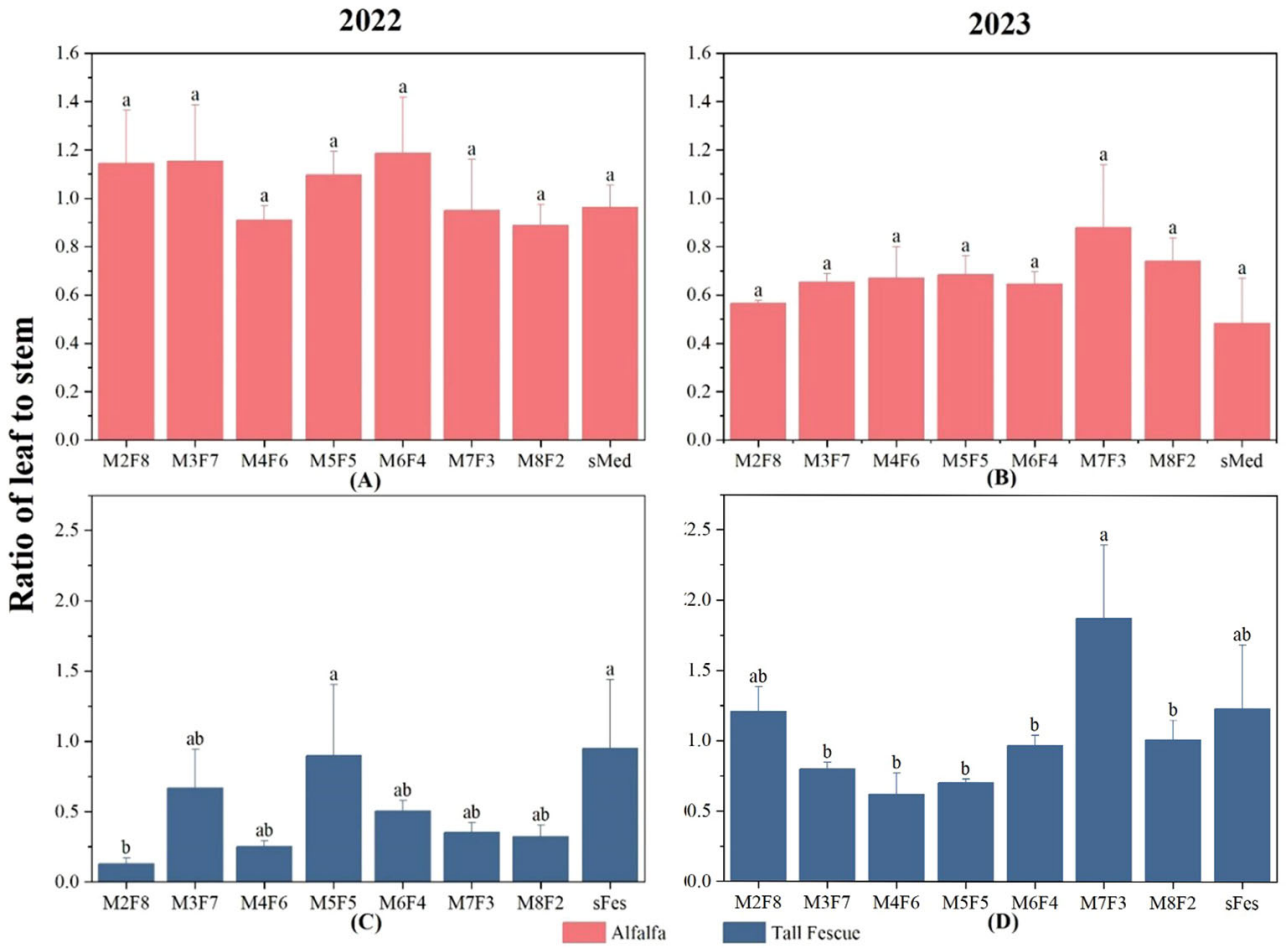
Stem-leaf ratio of plants under different inter-cropping ratios in 2022 and 2023. **(A)** Stem-leaf ratio of alfalfa under different inter-cropping modes in 2022. **(B)** Stem-leaf ratio of alfalfa under different inter-cropping modes in 2023. **(C)** Stem-leaf ratio of tall fescue under different inter-cropping modes in 2022. **(D)** Stem-leaf ratio of tall fescue under different inter-cropping modes in 2023.

In the first year, the CR of alfalfa in all intercropped treatments was greater than that of fescue, with the highest CR at M8F2 (**Figure 3**). In addition, alfalfa was the dominant crop. In the second year, the CR (**Figure 3**) of alfalfa showed a downward trend with increased alfalfa proportion, while the competitiveness of tall fescue was increased. The competitiveness of tall fescue was the greatest at M8F2.

### 3.2 Stem-leaf ratio of plants in the different inter-cropping treatments

There was no significant difference in the stem-leaf ratio of alfalfa in 2022. When the ratio of alfalfa to tall fescue was greater than 6, for the alfalfa-tall fescue mixtures under M7F3 and M8F2, the leaf proportion increased compared with that of M6F4, while the stem-leaf ratio decreased by 20% relative to M6F4 (**Figure 3**). With tall fescue, the stems to leaves ratio in M2F8 and M4F6 was significantly lower than in the other treatments (**Figure 3**). From M5F5, as the ratio of tall fescue decreased, the stems-to-leaves ratio decreased by 50%, and the leaves increased. In 2023 (**Figure 3**), the stem-leaf ratio of alfalfa was significantly different across the different treatments. In addition, the stem-to-leaf ratio of M7F3 was significantly higher than that of monoculture by 50%. In 2022, the stem-leaf ratio of tall fescue under M7F3 treatment was significantly lower than that of monocropping by 42%. Collectively, the stem-leaf ratio of alfalfa in intercropped plots was increased by about 20% compared to the monoculture. The stem-leaf ratio of tall fescue decreased first and then increased with the decrease of inter-cropping ratio. Notably, the stem-leaf ratio of M7F3 was significantly higher in the tall fescue treatment than in the other treatments. In addition, it was 51.9% higher than monoculture. Besides, it was 51.9% higher than the monoculture.

### 3.3 Effects of different inter-cropping ratios on soil water-soluble salt content

**Figure 4:**
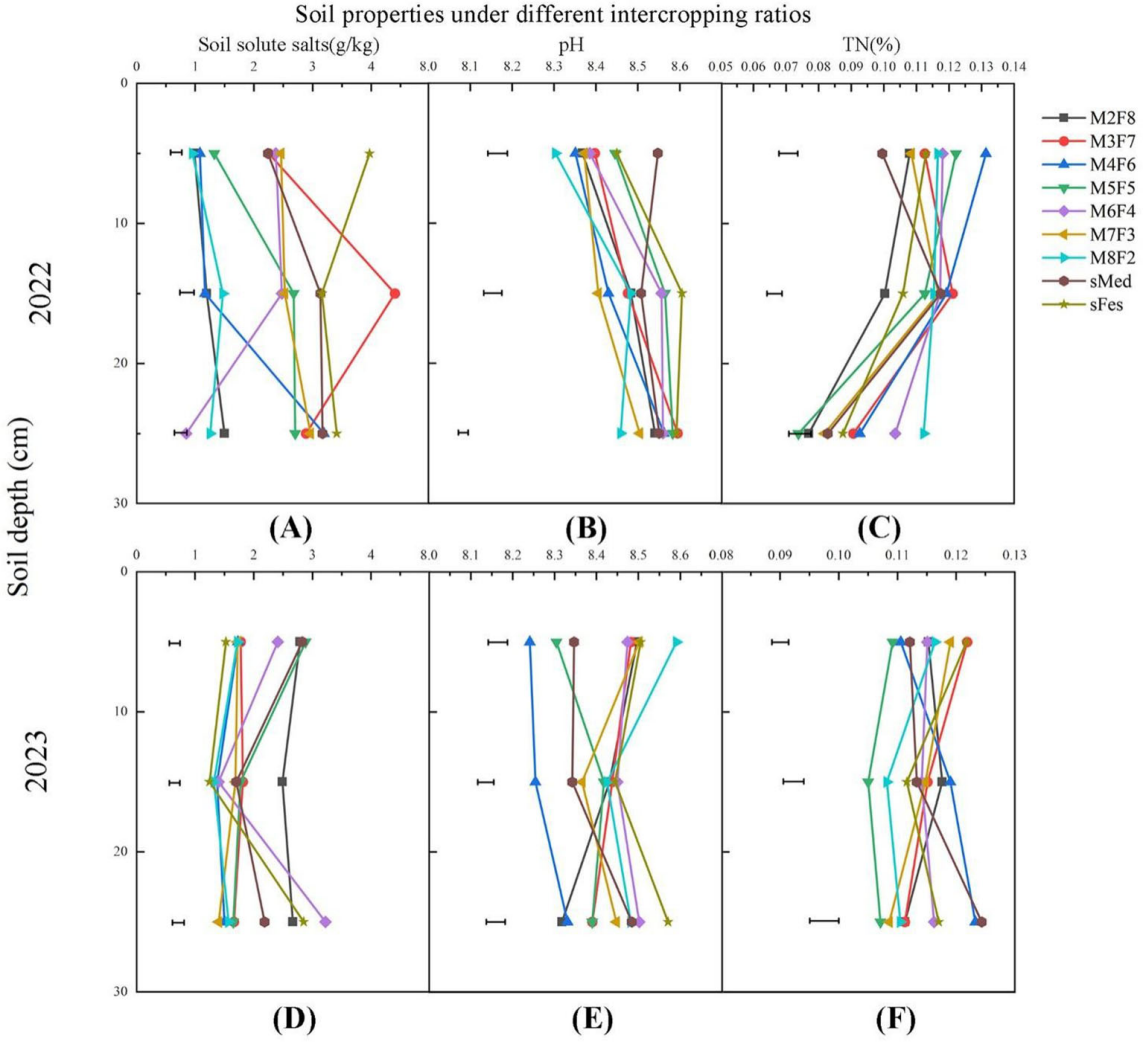
Soil soluble salts, pH, and total nitrogen (TN) of 0-30cm soil under different inter-cropping ratios in 2022 and 2023. Error bars represent LSD values. **(A)** Soil solute salts under different inter-cropping modes in 2022. **(B)** pH under different inter-cropping modes in 2022. **(C)** TN under different inter-cropping modes in 2022. **(D)** Soil solute salts under different inter-cropping modes in 2023. **(E)** pH under different inter-cropping modes in 2023. **(F)** TN under different inter-cropping modes in 2023.

**Figure 4** shows significant differences in the soil salt content under different inter-cropping ratios. Additionally, soil salinity decreased in 2023 compared with 2022. In the first year (2022, **Figure 4**), the soil salt content in the 0-10cm of intercropped treatments and SMed was significantly lower than that of sFes. Besides, the soil soluble salts in M2F8, M4F6, M5F5, and M8F2 were significantly lower than in the other inter-cropping treatments (**Figure 4**). At 10-20cm (**Figure 4**), the soil soluble salts were the lowest in M2F8, M4F6, and M8F2 and the highest in M3F7, higher than in the monoculture treatments. Besides, M2F8, M6F4, and M8F2 (**Figure 4**) had the lowest water-soluble salt content in the 20-30cm, and the salt content was not reduced in the other inter-cropping treatments compared to the monoculture treatments.

In the second year (2023), the soil soluble salts in the 0-10cm in sFes were significantly lower than in the other treatments. In the 10-20cm, the soil salt content of M2F8 was 2.663 g/kg, which was significantly higher than other treatments, and M8F2 and sFes were the lowest, 1.437 g/kg and 1.453 g/kg. At 20-30cm, the highest soil salt content was recorded in M6F4, and the soil salt contents in M3F7, M4F6, M5F5, M7F3, and M8F2 were significantly lower than the other treatments, including the monocultures. Compared to 2022, the soil salt content in M3F7, M7F3, and sFes in 2023 decreased by 21, 29.6, and 61.4%, respectively. Across all treatments, except M2F8, the water-soluble salt contents in 10-20cm and 20-30cm were decreased by 30-50% in 2023 compared to 2022.

### 3.4 Effects of the different inter-cropping ratios on soil pH

**Figure 5:**
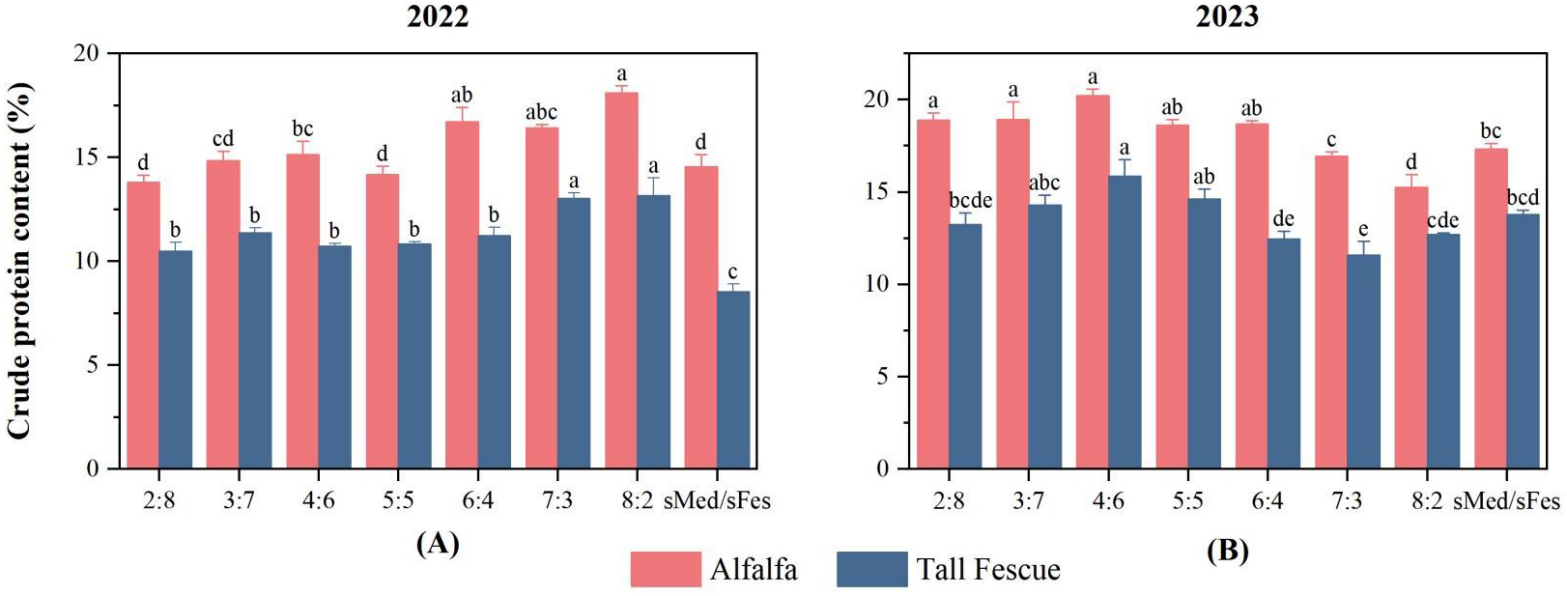
Crude protein content of plants under different inter-cropping ratios in 2022 and 2023. **(A)** Crude protein content under different inter-cropping patterns in 2022. **(B)** Crude protein content under different inter-cropping patterns in 2023.

There were significantly differences on soil pH among the different inter-cropping ratio treatments (**Figure 5**). In the first year, the soil pH at the 0–10 cm soil depth in M8F2 was the lowest and was significantly lower than sMed (**Figure 4**). At 10-20cm, the lowest soil pH was recorded in M7F3 and M8F2 at 20-30cm. In the second year, the lowest soil pH at 0-20cm was recorded in M4F6, which was significantly lower than the other treatments. Interestingly, there was no significant difference in pH in other. Compared to the first year, the average pH at 0-10cm was decreased by 1.3% and 1.6% in M4F6 and M5F5, respectively, and by about 1-2% in 10-20cm and 20-30cm.

### 3.5 Effects of the different inter-cropping ratios on the soil TN

Significant differences in the soil nitrogen content at 0-10cm existed between the different inter-cropping ratios in the first year (2022, **Figure 4**). Notably, the soil TN content was significantly higher in the inter-cropping than in the mono-cropping. The highest TN content was recorded in M4F6 and was the lowest in the controls. In the 10-20cm, the soil nitrogen content in M2F8 was significantly lower than that of other treatments, and M3F7 and M4F6 had the highest nitrogen content. In the 20-30cm, M8F2 had the highest soil TN content.

In the second year, the highest and lowest nitrogen content was recorded at 0-10cm in M3F7 and SMed, respectively. There was no significant change in the total nitrogen content in the deep soil. However, the nitrogen content in the 0-10cm was higher than that in the 10-20cm and 20-30cm. Compared to 2022, the TN contents in 0-10cm were increased in M2F8, M3F7, sFes, and SMed, and decreased in the other treatments compared to 2023. At the same time, the TN contents in M2F8 and sFes were increased at 10-20cm. At 20-30cm, the TN contents were increased by about 30%-40% in all treatments.

### 3.6 Effects of different inter-cropping ratios on plant crude protein

In 2022, alfalfa showed significantly higher crude protein content than tall fescue (**Figure 5**). Among the different inter-cropping ratios, the M8F2 mixture resulted in the highest crude protein content for alfalfa, which was 24% higher than sMed. For tall fescue, the M7F3 and M8F2 showed significantly greater crude protein content compared to others, with the highest value observed in M8F2 54% increase relative to sFes.

In 2023, the crude protein content of alfalfa and tall fescue with different intercropping ratios was generally higher than that of the crude protein in 2022 (**Figure 5**). Alfalfa maintained significantly higher crude protein levels compared to tall fescue. The alfalfa under the M4F6 treatment exhibited the highest crude protein content: it was 17% higher than that of the sMed treatment, and 24.6% higher compared to the crude protein content of alfalfa recorded in 2022. Notably, the same M4F6 ratio also yielded the highest crude protein content for tall fescue, which was 15% higher than sFes.

### 3.7 Effects of different inter-cropping ratios on relative feeding value

**Figure 6:**
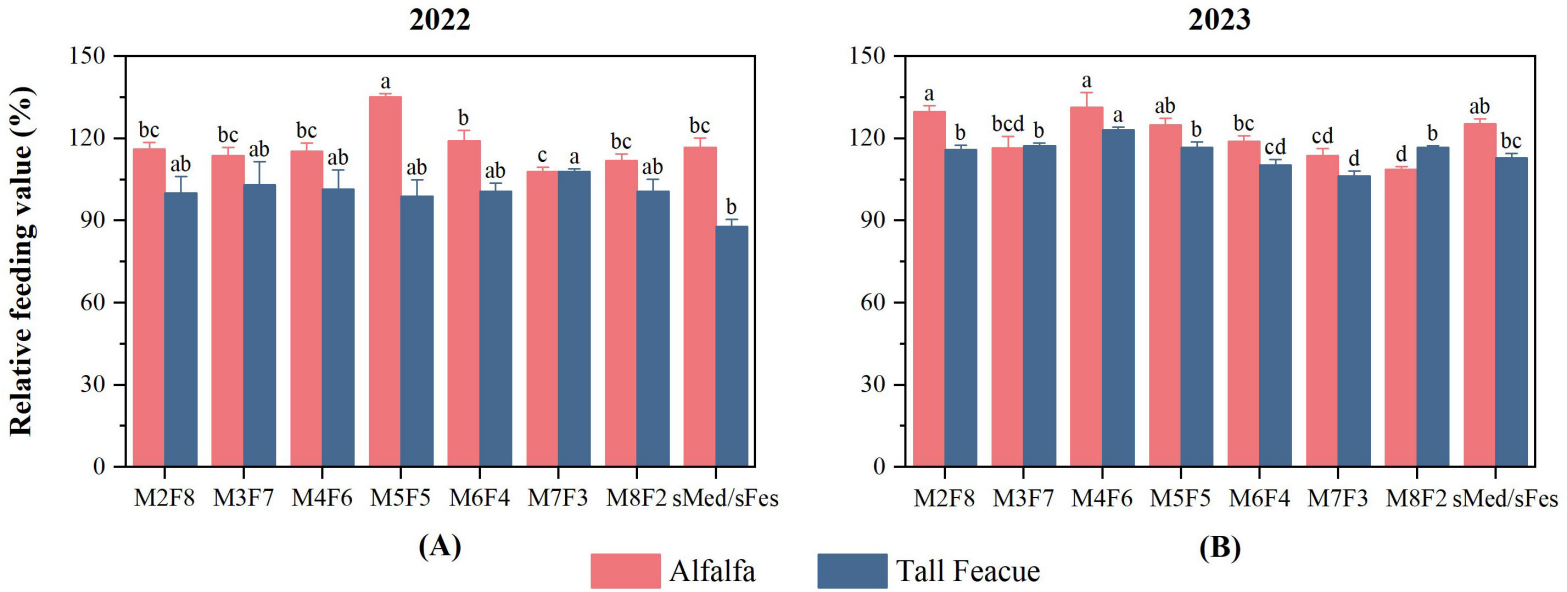
Relative feed value under different inter-cropping Ratios in 2022 and 2023. **(A)** Relative feed value under different inter-cropping patterns in 2022. **(B)** Relative feed value under different inter-cropping patterns in 2023.

In 2022, the relative forage intake value of tall fescue exhibited a trend of first rising and then falling, peaking at the M5F5 treatment. When the planting proportion of alfalfa exceeded 5, the crude fiber content of alfalfa increased while its palatability decreased, thereby leading to a reduction in the relative feeding value (**Figure 6**). Except for M7F3, the relative feeding value of tall fescue was higher than that of alfalfa. When the planting ratio of tall fescue exceeded M6F4, the relative feeding value of tall fescue decreased significantly, reaching the lowest value at M8F2. The relative feeding value of alfalfa was relatively stable before M6F4 without significant difference, reaching the maximum value at M3F7, greatly decreasing at M6F4 and reaching the minimum value at M8F2.

In 2023, the relative feeding value of alfalfa has increased compared with 2022 (**Figure 6**). Except for M3F7 and M8F2, the relative feeding value of alfalfa is higher than that of tall fescue. Among them, the relative feeding value of M2F8 and M4F6 is significantly higher than others. After M4F6, the relative feeding value of alfalfa shows an overall downward trend. The relative feeding value of tall fescue increased first and then decreased, and reached the maximum value at M4F6, which was significantly higher than others.

### 3.8 Correlation between yield traits and soil indexes under different inter-cropping ratios

**Figure 7:**
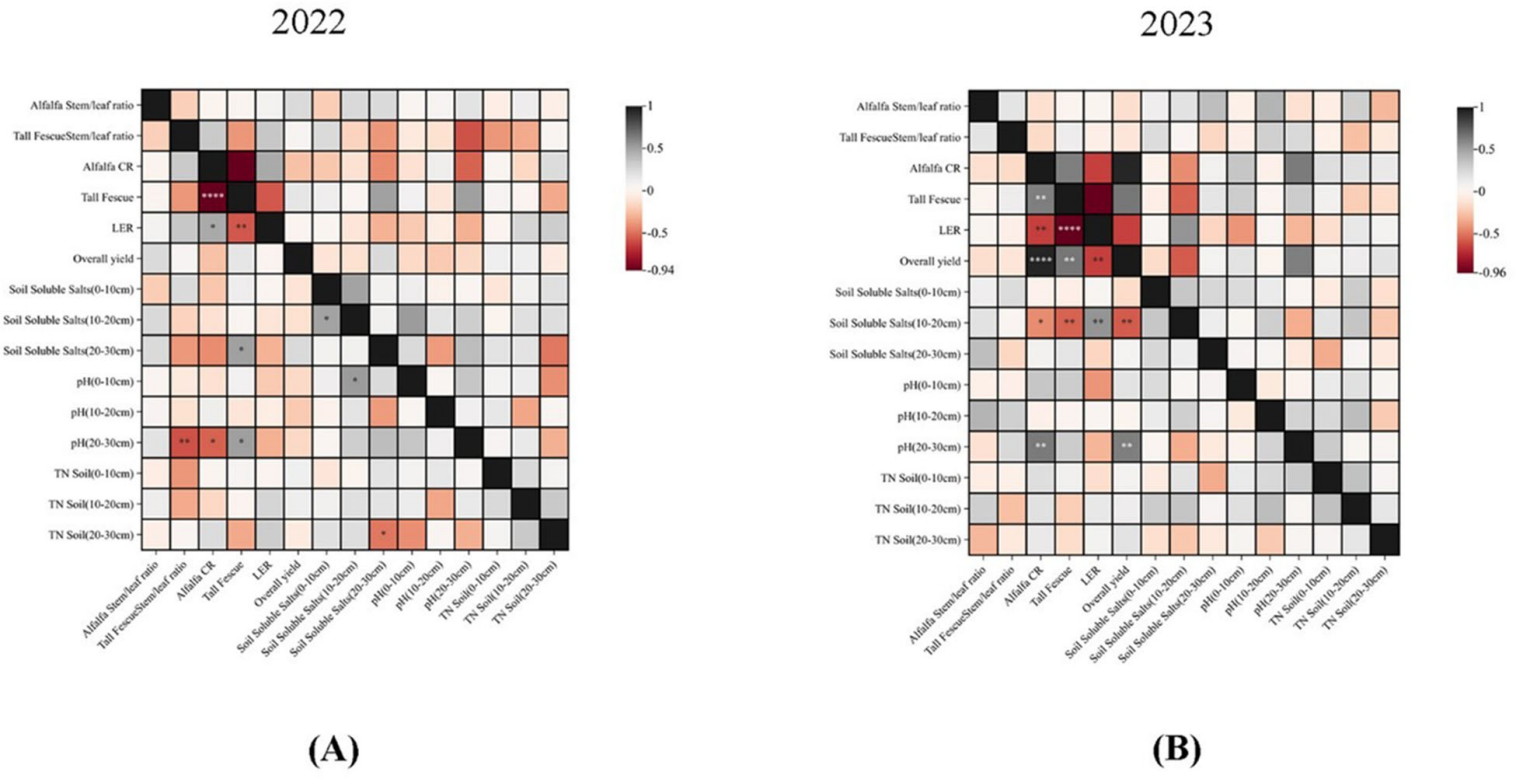
Pearson correlation analysis of plant yield, competition ratio (CR), land equivalent ratio (LER), stem-leaf ratio, 0-30cm soil soluble salts, pH, and TN under different inter-cropping ratios. Correlation R2 is represented by color, dark color represents strong correlation, light color represents weak correlation. **(A)** Correlation between 0-30cm chemical properties and plant traits under different inter-cropping modes in 2022. **(B)** Correlation between 0-30cm chemical properties and plant traits under different inter-cropping modes in 2023.

Pearson correlation analysis (**Figure 7**) revealed that the soil chemical indexes were correlated with the plant CR, stem-leaf ratio, and yield under different inter-cropping ratios. In the first year, soil pH was positively correlated with the soil salt content. However, the nitrogen content was negatively correlated with the soil salt content. In addition, the stem-leaf ratio of alfalfa was positively correlated with yield and CR, implying that alfalfa was the dominant species at this time and the key driver of the total yield. The higher the stem-leaf ratio, the lower the soil pH. Besides, the alfalfa ratio was negatively correlated with the salt content.

## 4 Discussion

### 4.1 Effect of inter-cropping ratio on above-ground biomass

This study revealed that the plant biomass from the different inter-cropping treatments in saline soil differed significantly. This result is similar to the results of Yang et al (Yang et al., 2017), inter-cropping improved the overall yield. Under resource-limited conditions, plants demonstrate better physiological performance than when resources are abundant (Wang et al., 2021). It is worth noting that the plant yield in the second year was stable and higher than that in the first year, possibly due to the increased competitiveness between the two grasses in the second year and improved resource utilization efficiency, thus greatly increasing the yield. Besides, the overall productivity in the first year depended on the alfalfa yield. Generally, the more stable the yields of intercropped plants, the more stable the productivity of the inter-cropping system (Wu et al., 2022). In an inter-cropping system, the intercropped species compete for resources. Higher alfalfa proportions and inter-cropping strip widths increased productivity, with higher alfalfa proportions producing higher yields in this study. Thus, the yield increase of one species often comes at the cost of yield loss of other intercropped species, given the dynamic competition (Justes et al., 2021).

In this study, the competitiveness of the two inter-cropped species between the first and second year was different, and the inter-cropping ratio affected the species competitiveness. For example, M6F4, M7F3, and M8F2 had the same CR, with a higher LER than other treatments. In addition, their land use efficiency was high, with strong CR in both or one species, which ensures the population yield. Thus, different inter-cropping ratios alter the spatial composition of the above-ground growth, and the plant CR is different, consistent with the findings of other researchers (Bing et al., 2022), who appropriately increased the inter-cropping width to reduce corn competition. Another study showed that alfalfa in the alfalfa/corn inter-cropping system has a higher CR (Zhang et al., 2011; Wu et al., 2023). In inter-cropping systems, the total yield is higher than in monoculture systems because of the complementarity of the two plant species. Even if the yield of one plant decreases, the yield of the other plant increases, which is determined by the size of interspecific and intraspecific competition. In this study, altering the inter-cropping ratio improved the land use efficiency. For example, the above-ground spatial distribution of plants in M6F4, M7F3, and M8F2 regulated the intraspecific and interspecific competition. Alfalfa was the important component supporting the total yield between the two crops. Besides, inter-cropping was more high-yielding and stable than the monoculture treatments, increasing the plant yield within the competitive range. Changing the intercropping ratio increased the crude protein content and relative forage value of alfalfa and tall fescue, but the cellulose content and palatability of alfalfa and tall fescue decreased when the intercropping ratio increased more than M8F2 ratio (Liu et al., 2025). In 2022, alfalfa crude protein reached its maximum at M8F2. In 2023, the crude protein content of alfalfa reached the maximum value at M4F6, and then decreased with the increase of alfalfa proportion, which may be related to soil nutrients in newly planted grassland (Smutny et al., 2025). At the same time, compared with the relative feeding value of forage in 2022, the relative feeding value of forage in 2023 has increased, which may be related to the neutral detergent fiber and acidic detergent fiber of forage. With the increase of perennial forage over time, the content of neutral detergent fiber and acidic detergent fiber decreases, which greatly increases the relative feeding value of forage (Sanson and Kercher, 1996).

In the wheat/maize inter-cropping system, different row ratios altered the above-ground light competition and improved the light utilization efficiency (Wang et al., 2017). In another study, Maize/legume inter-cropping systems were demonstrated to increase leaf and dry matter yields (Fu et al., 2023). In the maize/peanut inter-cropping system, the yield response is significantly altered by changing the row ratio. Notably, efficiently increasing the row width increases the light interception on the ground (Raza et al., 2019). Besides, the row configuration in the maize/soybean inter-cropping system alters the photosynthetic parameters (Li et al., 2021). Herein, with the increase in the alfalfa ratio, the alfalfa leaf ratio was significantly increased, and the stem-leaf ratio was decreased. Similarly, with a tall fescue ratio greater 5, the stem-leaf ratio was increased, implying that the ratio of leaves was increased with the increase in plant ratio and enhanced the light acquisition, which may be the reason for the increased yield at specific inter-cropping ratios.

### 4.2 Effects of different alfalfa-tall fescue inter-cropping ratios on soil chemical properties

inter-cropping significantly alters the soil hydraulic properties and nutrients, optimizing the soil structure (Lei et al., 2023). This study showed significant differences in the soil salinity under different inter-cropping ratios. Overall, inter-cropping significantly reduced the salt content in the 0-10cm and the soil salt content reduced from 2022 to 2023. Correlation analysis further revealed that the plant stem-leaf ratio was directly proportional to the salt content in surface soil. inter-cropping increases the coverage rate, reducing the accumulation of evaporated salt in the surface layer (Hu et al., 2020). The deep-rooted grass in the inter-cropping system absorbs deep soil moisture and reduces the salt accumulation to the surface caused by water evaporation. Alfalfa is one of the main plants capable of reducing the soil salt content, with its rooting depth reaching 120cm in the second year of growth (Wang et al., 2021). Additionally, inter-cropping may alter the alfalfa root architecture (Liu et al., 2023) and enhance the soil water conductivity (Secco et al., 2023), reducing salt accumulation in the surface soil layer.

Water transport by roots is an important mechanism for water utilization and complementation in inter-cropping systems (Sekiya et al., 2010). In one study of inter-cropping systems, a higher proportion of alfalfa had a higher potential for salt uptake. Herein, the reduced soil salinity in M3F7 and M4F6 may be due to more rows of Festuca arundinacea, which resulted in more roots in the surface layer, more soil pores, and increased water conductivity. However, the reduced soil salinity in M7F3 can be attributed to more rows of alfalfa, which absorbed more salt and had a higher coverage area, reducing the soil salt accumulation. In this study, changes in the inter-cropping ratio reduced the water-soluble salt content of the soil and changed the soil structure. Besides, the increased root growth in the second year could have also contributed to the reduced soil salinity.

In this study, inter-cropping increased the total nitrogen content of the soil, which was significantly higher than that of the mono-cropping treatment, and there were significant differences between different inter-cropping ratios, consistent with the results of (Zhang et al., 2019). In inter-cropping systems, the increase in nitrogen fixation capacity of leguminous plants is related to the complementary effect (Yi et al., 2023). The niche complementarity effect shows that the differences in the space and time between different species cause different access to resources, resulting in interspecific promotion (Loreau et al., 2001). In the alfalfa/triticale inter-cropping system, the inter-specific promotion effect improved the node-fixing ability of alfalfa and the yield and quality of crops (Zhao et al., 2020). Alfalfa and tall fescue have different root distributions and complementary niches. Besides, alfalfa nodules fix nitrogen, transfering nitrogen from the atmosphere to gramineae (Nygren and Leblanc, 2015). Similarly, another study showed that more nitrogen was obtained from legumes and inter-cropping systems (Tilman et al., 2001; Bessler et al., 2012). In this study, the M4F6 ratio resulted in the highest soil nitrogen content, with the lowest nitrogen content across the three soil layers in the alfalfa monoculture plot. This can be attributed to the alfalfa monoculture inhibiting nitrogen fixation due to the increased fixed nitrogen concentration while inter-cropping alfalfa promoted nitrogen fixation ability due to the decrease of nitrogen concentration due to nitrogen transfer.

## 5 Conclusions

M6F4, M7F3, and M8F2 inter-cropping ratios yield the highest productivity. Concurrently, the M6F4 inter-cropping ratio exhibited the highest crude protein content and relative feeding value among all treatments, indicating its superior forage quality. The inter-cropping ratios alter the competitive ability of plants, which leads to different plant growth strategies, such as altering the stem-leaf ratio, spatial structure above ground, and soil structure below ground. With alfalfa and tall fescue inter-cropping, M2F8, M4F6, M5F5, and M8F2 inter-cropping ratios significantly reduce the soil salinity and pH and increase the TN concentration. Therefore, in intercropping, appropriately increasing the proportion of alfalfa can improve the utilization efficiency of mild saline-alkali land, produce higher yield and improve soil chemical properties. These results have a guiding significance for the utilization of saline land.

## Data Availability

The original contributions presented in the study are included in the article/supplementary material. Further inquiries can be directed to the corresponding authors.
